# Kawasaki Disease Shock Syndrome With Progressive AV Block and Atrioventricular Dissociation: Case Report

**DOI:** 10.1111/jpc.70210

**Published:** 2025-10-07

**Authors:** Chengjun Dai, Jiao Chen, Yutong Chen, Yingchao Ying, Yanlan Fang, Zihao Yang, Chunlin Wang

**Affiliations:** ^1^ Department of Pediatrics, The First Affiliated Hospital, College of Medicine Zhejiang University Hangzhou Zhejiang China; ^2^ National Center for Clinical Research in Child Health and Disease Hangzhou ZhejiangHangzhou China; ^3^ Children's Hospital Zhejiang University School of Medicine Hangzhou Zhejiang China

## 1 Introduction

Kawasaki disease (KD) is an acute autoimmune vasculitis that primarily affects small to medium‐sized arteries and can lead to multiple organ dysfunction. The classic clinical manifestations of KD include fever, conjunctival injection, cervical lymphadenopathy, polymorphous rash and oropharyngeal changes. In developed countries, KD is the leading cause of acquired heart disease in children. Without treatment, approximately 15%–25% of affected children may develop coronary artery aneurysms. Administering intravenous immunoglobulin (IVIG) within the first 10 days significantly improves the prognosis. However, some patients may present with atypical features, complicating early diagnosis.

On rare occasions, patients with KD may experience hypotension, heart failure and reduced organ perfusion. In 2009, Kanegaye et al. defined this hemodynamically unstable form of KD as Kawasaki disease shock syndrome (KDSS) [1]. KDSS is a severe complication, occurring in 1% to 7% of KD cases [2]. While the mechanism and pathophysiology of KDSS remain unclear, a cytokine storm and capillary leak are speculated to be the underlying causes [3]. Previously reported cardiac damage in KDSS has been primarily attributed to systolic dysfunction or coronary artery dilation [4], with few cases involving abnormalities in the cardiac conduction system. Here, we report a case of KDSS with a severe second‐degree atrioventricular block and a reduced ventricular rate.

## 2 Case Presentation

A 10‐year‐old girl initially presented with cervical lymphadenopathy and fever. She had no prior medical or family history. On the third day of illness, she was admitted to the paediatric ward with a diagnosis of lymphadenitis. Her symptoms included swollen, painful cervical lymph nodes, elevated skin temperature in the neck and a high fever (40.4°C). At the time, KD was not suspected as she lacked typical features such as a strawberry tongue, cracked lips, or conjunctivitis. Laboratory tests showed a C‐reactive protein (CRP) level of 49.9 mg/L, a white blood cell count of 12.54 × 10^9^/L, a haemoglobin level of 134 g/L and a platelet count of 184 × 10^9^/L. Her sodium level was 133 mmol/L, potassium was 4.24 mmol/L and transaminase levels were normal. PCR tests for viruses, including SARS‐CoV2, influenza and adenovirus, were negative. The bacterial culture result was also negative. An electrocardiogram revealed a normal sinus rhythm. The preliminary diagnosis was cervical lymphadenitis and ceftriaxone was administered to treat the infection.

However, by the fifth day, her fever persisted and her CRP level had risen to 118.11 mg/L. As a result, her antibiotic regimen was changed to sulbactam and cefoperazone. On the sixth day, her condition worsened. Her fever remained above 39°C, and she developed abdominal pain, oliguria and hypotension (blood pressure of 62/31 mmHg). Immediate fluid resuscitation and administration of dopamine and norepinephrine were initiated. Blood and cardiac tests were performed, with echocardiography showing an ejection fraction of 60% and no coronary artery dilation, with mild mitral regurgitation. Suspecting septic shock, we started treatment with vasoactive drugs, linezolid and meropenem. A lung CT scan revealed pneumonia and pleural effusion. Kidney function tests indicated acute kidney injury, with blood urea nitrogen at 14.16 mg/dL and serum creatinine at 249 μmol/L.

**FIGURE 1:**
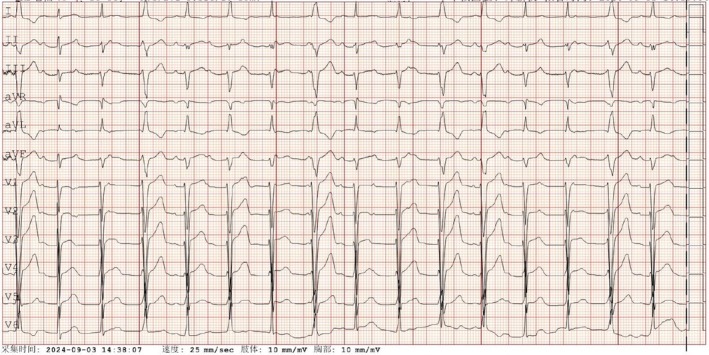
Electrocardiogram showed frequent ventricular premature beats.

Her blood pressure improved, but she developed arrhythmia, with an electrocardiogram showing premature beats, atrioventricular block and a left‐sided heart axis. (Figure 1). Gradually, signs of KD began to emerge, including conjunctival injection and a strawberry‐like tongue. Based on these findings, KDSS was diagnosed. Intravenous immunoglobulin and aspirin were administered on the seventh day to control inflammation. Her fever subsided, and kidney function gradually improved. The patient's blood test results are presented in Table 1.

**TABLE 1:** Main laboratory data of the patient.

	Day 3	Day 5	Day 6	Day 7	Day 8	Day 12
WBC (×10^9^)	12.54	9.7	10.79	15.25		16.46
NEU (×10^9^)	11.24	9.26	10.30	14.27		13.82
LYM (×10^9^)	0.5	0.22	0.29	0.63		2.20
HB (g/L)	134	129	106	114		97
PLT (×10^9^)	184	166	144	179		286
CRP (mg/L)	49.9	118.11	116.17	124.83		7.18
ALT (U/L)	8		33	28	27	65
AST (U/L)	25		47	40	30	56
Scr (μmoI/L)	53		249	154	57	33
BUN (mmol/L)	5.88		14.16	11.79	9.34	6.34
K (mmol/L)	4.24	4.22	4.28	3.44	3.31	
Na (mmol/L)	133	131	132	141	136	
Cl (mmol/L)	97	94	97	102	96	
CK (U/L)	44		53	35	17	16
CK‐MB (U/L)	23		36	17	13	14
TNI (ng/mL)			0.019	0.049	0.012	
d‐Dimer (μg/L)	370		4490	7620	5780	1970
Ferritin (μg/L)			1156.6	1449.4	1081.0	1168.4

Abbreviations: ALT = alanine aminotransferase, AST = aspartate aminotransferase, BUN = blood urea nitrogen, CK = creatine kinase, CK‐MB = creatine kinase‐MB, CRP = C‐reactive protein, HB = haemoglobin, LYM = lymphocyte, NEU = neutrophils, PLT = platelet, Scr = serum creatinine, TNI = troponin‐I, WBC = white blood cell.

**FIGURE 2:**
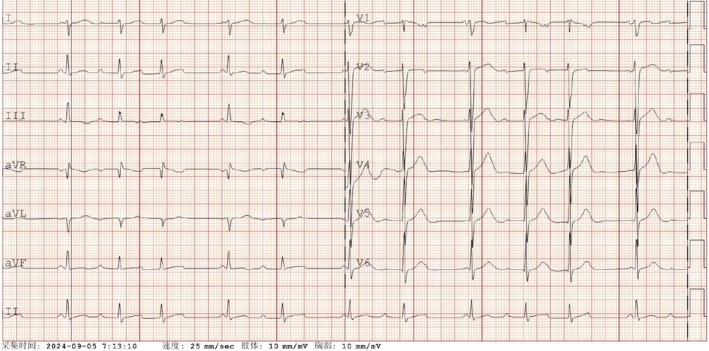
Electrocardiogram showed one type of second‐degree atrioventricular block.

**FIGURE 3:**
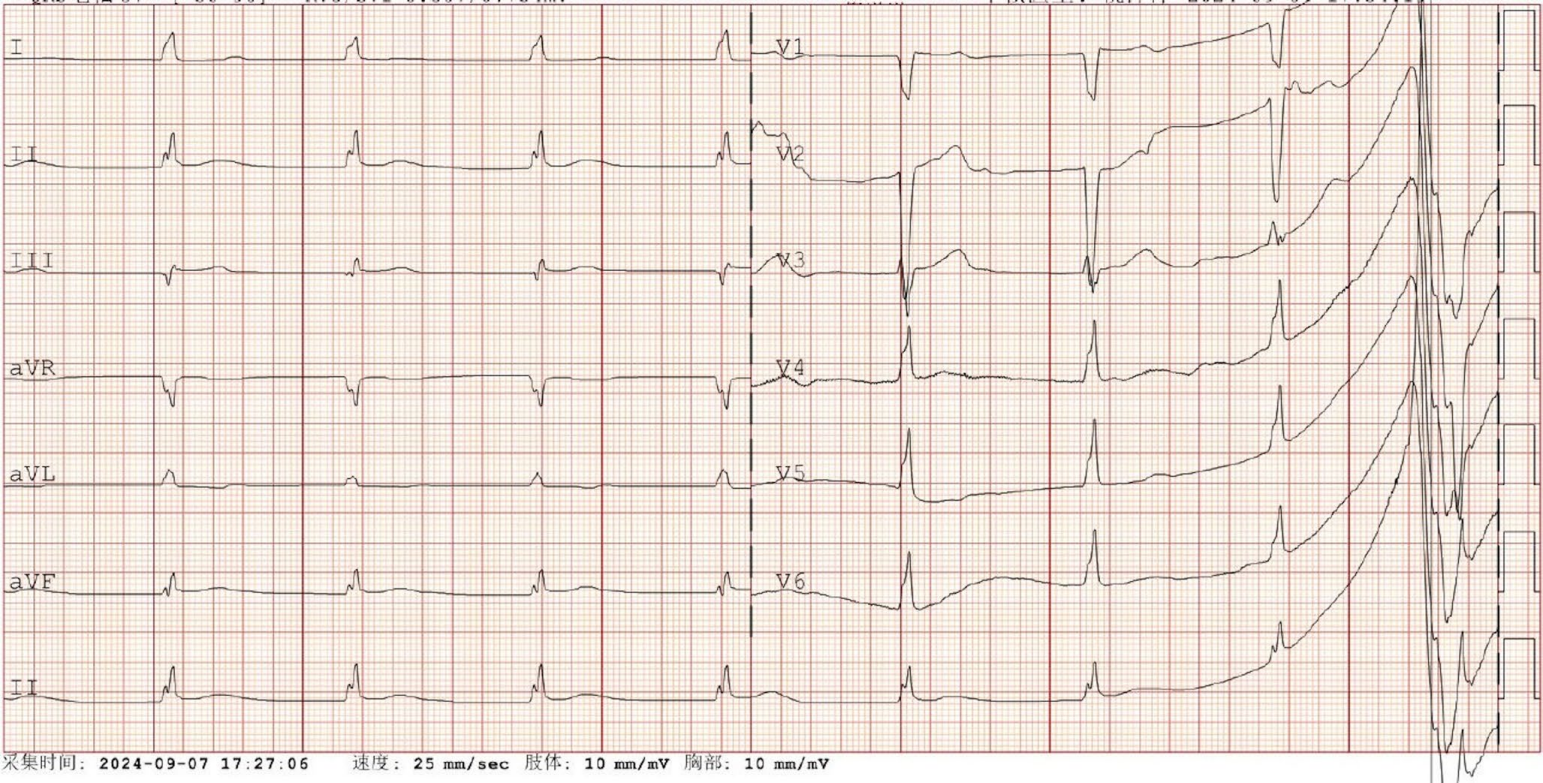
Electrocardiogram showed sinus arrest and ventricular escape.

On the sixth day of illness, new abnormal symptoms emerged. The patient developed an unusual arrhythmia, initially presenting as frequent ventricular premature beats. Cardiac tests revealed elevated levels of troponin (0.049 ng/mL) and BNP (> 35 000 ng/L), suggesting myocardial injury due to KDSS. Following the administration of intravenous immunoglobulin (IVIG), troponin levels decreased to 0.012 ng/mL. However, an electrocardiogram showed complete atrioventricular block (Figure 2). By the eighth day, the patient's ventricular rate slowed significantly, with the lowest heart rate dropping to 40 beats per minute. The electrocardiogram showed atrioventricular dissociation (Figure 3). Given the severity of the arrhythmia, intravenous steroid pulse therapy (10 mg/kg) was administered on the eighth and ninth days, along with isoproterenol to increase the heart rate. Simultaneous 24‐h Holter monitoring demonstrated 16 premature atrial contractions, intermittent first‐degree atrioventricular block, 7 episodes of second‐degree type I atrioventricular block, 435 ventricular escape beats and intermittent QT interval prolongation. Fortunately, by the 11th day, her heart rate stabilised into a sinus rhythm of 80 beats per minute and remained consistent thereafter.

Serial echocardiographic examinations revealed a small amount of pericardial effusion and dilation of the left coronary artery to 0.34 cm (*Z* = 1.1) on the 11th day. The inner diameter of the left anterior descending branch measured 0.23 cm (*Z* = 0.1), the circumflex branch was 0.19 cm (*Z* = −0.6) and the proximal right coronary artery opening measured 0.28 cm (*Z* = 0.4), while the distal right coronary artery diameter was 0.36 cm (*Z* = 3.2). No further coronary artery dilation was observed during subsequent follow‐ups. The patient remained asymptomatic while continuing aspirin therapy and undergoing gradual tapering of steroid treatment. At follow‐up 9 months after discharge, the echocardiogram showed no coronary artery aneurysm or dilation, with the LVEF 64%.

## 3 Discussion

We describe a rare case of a young girl with KD who developed KDSS accompanied by severe arrhythmia. Initially misdiagnosed due to her atypical presentation, her first symptoms were fever and cervical lymphadenitis. Antibiotic treatment proved ineffective, and shock occurred on the sixth day of illness. It was only after the appearance of strawberry tongue and conjunctivitis that KD was suspected. Diagnosing KD early is challenging when the typical clinical manifestations have not yet appeared. In this case, the diagnosis remained uncertain until the onset of shock. In clinical practice, KDSS is often misdiagnosed as septic shock [5], as both conditions present with peripheral circulatory dysfunction, hypotension, elevated CRP and increased interleukin‐10 (IL‐10) [6]. However, KDSS is more likely to involve myocardial dysfunction and coronary artery dilation [7]. The exact cause of KDSS remains unknown, but it is often associated with capillary leakage, cytokine storms and myocardial dysfunction.

It is also important to distinguish KDSS from multisystem inflammatory syndrome in children (MIS‐C), as they share nearly identical clinical features. However, MIS‐C is linked to SARS‐CoV‐2, while KDSS tends to occur more frequently in Asian countries. In our case, the girl was not diagnosed with MIS‐C, as her test for SARS‐CoV‐2 was negative. Moreover, MIS‐C is more commonly documented in Western nations (Europe and the Americas), while KDSS exhibits a higher incidence in Asian countries. An additional distinguishing feature is that MIS‐C is more frequently associated with macrophage activation syndrome (MAS) [8]. The clinical and laboratory features of our case align more closely with the diagnostic criteria for KDSS than for MIS‐C.

KDSS can lead to myocardial dysfunction, typically indicated by a reduced ejection fraction. To our knowledge, this is the first reported case of KDSS associated with abnormal cardiac conduction function. It is reported that approximately 80% of KDSS patients show elevated levels of creatine kinase‐MB and troponin‐T [9], suggesting myocardial cell injury. In one case from Japan, a myocardial biopsy revealed mild fibrosis and infiltration of the interstitial myocardium by macrophages and T‐lymphocytes [10]. However, other studies have shown that elevated troponin levels are not always common [4]. Reports of injury to the cardiac conduction system are even rarer. In our case, the electrocardiogram initially showed complete atrioventricular block, which progressed to AV dissociation. After the administration of high‐dose IVIG and intravenous steroid pulse therapy, the patient's sinus rhythm was restored, avoiding the need for artificial pacemaker implantation. The electrocardiogram changes corresponded with the treatment course, leading us to conclude that the arrhythmia was a manifestation of KDSS. The early use of glucocorticoids in this case offers valuable insights for future management of similar cases.

The incidence of KDSS is relatively low, and some studies have developed logistic regression prediction models for analysis [11]. Unlike typical KD, patients with KDSS often present with lower platelet counts. This decrease may be linked to platelet consumption due to abnormally active coagulation, a possibility supported by elevated D‐dimer levels. Additionally, another study identified decreased serum sodium as an independent risk factor for KDSS [7]. Our patient generally aligned with these predictive models; however, she differed from previous reports in that her transaminase and myocardial enzyme levels remained normal. These variations highlight the heterogeneity amongst different KDSS patients.

Coronary artery abnormalities are more likely to occur in KDSS. In our case, the patient developed dilation of the distal right coronary artery (*Z* = 3.2). Fortunately, there was no further dilation observed during follow‐up. Compared to typical KD, KDSS patients often exhibit a higher failure rate following initial IVIG treatment. One potential mechanism for IVIG resistance may be the insufficient levels of anti‐cytokine antibodies, which are unable to block excess cytokines. The early use of glucocorticoids may help mitigate this issue, as they can downregulate inflammatory mediators.

In conclusion, patients with KDSS may be misdiagnosed due to incomplete clinical manifestations. When anti‐infective treatment for children with septic shock proves ineffective, KDSS should be considered as a potential diagnosis. Furthermore, arrhythmia may serve as a primary manifestation of myocardial injury. Previous studies have also shown that early corticosteroid shock therapy (1000 mg/day for 17‐year‐old youth) can help improve the prognosis of KDSS [10]. In our case, 10 mg/kg methylprednisolone treatment was also effective. Early and appropriate dosing of IVIG and corticosteroids is essential for controlling disease progression and improving prognosis.

## 4 Learning Points

Atrioventricular block with a reduced ventricular rate and atrioventricular dissociation may represent another manifestation of myocardial injury in KDSS. Early immunosuppressive treatment, including steroid pulse therapy and IVIG, may alleviate symptoms and improve prognosis.

## Ethics Statement

This research protocol received approval from the Ethics Committee of the First Affiliated Hospital of Zhejiang University School of Medicine (IIT20250254A).

## Consent

The patient's parent provides his consent to submission, and we obtained written consent from the patient's parents. The consent that was obtained from all of the participants.

## Conflicts of Interest

The authors declare no conflicts of interest.

## Data Availability

The data that support the findings of this study are available from the corresponding author upon reasonable request.
